# From prophylactic mRNA vaccines against viral infections to therapeutic applications: regulatory science considerations for expanding mRNA platforms

**DOI:** 10.3389/fimmu.2026.2007961

**Published:** 2026-10-30

**Authors:** Sunghyun Yi, Eunhye Seo, Pil-Gu Park, Kee-Jong Hong

**Affiliations:** 1 Korea mRNA Vaccine Initiative, Gachon University, Seongnam, Republic of Korea; 2 Department of Health Policy and Management, General Graduate School, Gachon University, Seongnam, Republic of Korea; 3 Department of Microbiology, College of Medicine, Gachon University, Incheon, Republic of Korea; 4 Department of Life Sciences, Gachon University, Seongnam, Republic of Korea

**Keywords:** cancer immunotherapy, mRNA therapeutics, mRNA vaccines, platform technology, regulatory classification, vaccine regulation

## Abstract

Conventional non-amplifying messenger RNA (mRNA) and self-amplifying RNA (saRNA) medicines generally share a core linear RNA architecture—including a 5′ cap, untranslated regions, one or more open reading frames, and a poly(A) tail—and are commonly formulated with delivery systems such as lipid nanoparticles (LNPs) to enable transient intracellular expression of a target protein. Prophylactic mRNA vaccines are designed primarily to express infectious-disease antigens and generate protective immune memory, whereas mRNA therapeutics exert pharmacological effects intended to treat disease, suppress recurrence, replace proteins, modulate immunity, or deliver gene-editing enzymes. Thus, even products based on similar mRNA–LNP technology may have different regulatory classifications and evidence requirements according to their intended use and mechanism of action. The clinical and regulatory landscape has expanded beyond the first coronavirus disease 2019 vaccines to include respiratory syncytial virus and influenza mRNA vaccines, an influenza–coronavirus disease 2019 combination vaccine, an authorized saRNA vaccine, personalized cancer immunotherapies, protein-replacement therapies, *in vivo* cell programming, and gene-editing delivery systems. This review examines which prophylactic-vaccine evidence can support therapeutic development and where product-specific quality, nonclinical, clinical, and lifecycle evidence remains necessary. For prophylactic mRNA vaccines, major regulatory considerations include quality, immunogenicity, preventive efficacy, lot release, and safety surveillance after large-scale vaccination. For mRNA therapeutics, the principal issues include delivery to target tissues, function of the expressed product, repeat-dose safety, biodistribution, off-target risk, long-term follow-up, and patient-specific manufacture and traceability. In particular, although personalized mRNA cancer vaccines are commonly described as vaccines, they are closer in regulatory purpose to therapeutics that act on the basis of each patient’s tumor characteristics than to vaccines that prevent infectious disease. Antigen-selection algorithms, patient-specific sequence design, small-batch and multi-product manufacture, product–patient matching, clinical evaluation of combination strategies, manufacturing delays and analysis populations, and risk-based long-term postauthorization follow-up therefore require explicit regulatory consideration. Platform-level knowledge from prophylactic mRNA vaccine authorization can support therapeutic development, but regulatory questions may vary with each new product type; developers will need to track evolving regulatory instruments across jurisdictions and engage regulatory authorities early.

## 1 Introduction

The rapid deployment of prophylactic messenger RNA (mRNA) vaccines against coronavirus disease 2019 expanded clinical and regulatory experience with the platform. In parallel, therapeutic development has advanced in personalized cancer immunotherapy, protein replacement for rare metabolic diseases, and *in vivo* genome editing (1–5). Most of the regulatory experience with mRNA products, however, concerns vaccines designed to prevent viral infection; therapeutic products may differ in intended use, dosing, manufacturing scale, and benefit–risk profile. In this review, we categorize mRNA products into three groups: prophylactic mRNA vaccines; vaccine-form mRNA therapeutics, such as personalized (also termed individualized) cancer vaccines intended to induce treatment-directed antigen-specific immunity; and non-vaccine mRNA therapeutics, including protein or enzyme replacement, antibody or cytokine expression, and genome editing. This grouping is descriptive; how such products are classified in each jurisdiction is examined in the regulatory classification section.

Conventional non-amplifying mRNA and self-amplifying RNA (saRNA) products generally share a core linear RNA architecture comprising a 5′ cap, 5′ and 3′ untranslated regions (UTRs), one or more open reading frames (ORFs), and a poly(A) tail, and are commonly formulated with delivery systems such as lipid nanoparticles (LNPs) (1, 6, 7). The design priorities nevertheless differ. Prophylactic mRNA vaccines emphasize antigen expression and the formation of immune memory, whereas mRNA therapeutics may require sufficient expression of a therapeutic protein in the target tissue, control of expression duration, suitability for repeat administration, minimization of non-target exposure, and suppression of unwanted innate immune activation or deliberate immunomodulation consistent with the intended mechanism (2, 3). Engineered circular RNA (circRNA) is included here within the broader protein-coding RNA platform family but differs from the linear architecture described above: it is covalently closed and lacks the canonical 5′ cap and poly(A) tail, relies on cap-independent translation, and may support more persistent expression than linear mRNA (3). These structural and translational differences require product-specific justification when evidence from linear non-replicating mRNA or saRNA products is transferred.

Protein-replacement mRNA therapeutics act by enabling the body to produce a protein that is absent or functionally deficient. When the encoded protein is secreted, a systemic pharmacological effect may be achieved by attaining an adequate circulating concentration; when an intracellular enzyme is encoded, the product may be designed to restore metabolic function in the target tissue. High translation efficiency and control of immunogenicity during repeat administration are therefore central development challenges (2, 3).

Genome-editing mRNA therapeutics can be used to express editing proteins such as Cas-family nucleases, base editors, or prime editors transiently (1, 2, 5, 8). Although expression of the administered mRNA is transient, a therapeutic effect may persist when the expressed enzyme edits the target DNA. The nonintegrating nature of conventional mRNA and the durability of the resulting edit should therefore be evaluated separately. Co-delivery with guide RNA, delivery to the intended tissue, off-target risk, and the feasibility of repeat administration are major considerations.

Vaccine-form mRNA therapeutics share part of their mechanism with prophylactic mRNA vaccines because both induce antigen-specific immune responses, but the clinical purpose of the former is treatment rather than disease prevention (4, 9). A personalized mRNA cancer vaccine, for example, can be designed by incorporating multiple tumor-specific antigens into an mRNA sequence on the basis of neoantigens identified through genomic or transcriptomic analysis of an individual patient’s tumor (10). Expression and presentation of these antigens by antigen-presenting cells can induce tumor-specific CD4+ and CD8+ T-cell responses intended to eliminate residual tumor cells or reduce recurrence. Thus, the dosage form resembles a vaccine, but the pharmacological purpose is therapeutic.

Administration is a separate design axis: mRNA may be delivered directly *in vivo* or used to modify cells ex vivo. With direct *in vivo* administration, biodistribution to the target tissue and formulation characteristics strongly influence performance; ex vivo strategies may introduce mRNA into patient-derived cells, including T cells or dendritic cells, before the cells are administered. These features make the duration of pharmacological activity, target-tissue or target-cell selectivity, repeat-dose toxicity, immunogenicity, potential accumulation of delivery-system components, and functional testing of the expressed protein important development considerations (1–3, 7, 11).

Previous reviews have proposed platform-based approaches to mRNA product development and regulation, including transfer of common manufacturing and quality knowledge, adaptation of nonclinical and clinical evidence, and representative-product strategies for individualized neoantigen therapies (12, 13). A recent review of personalized cancer vaccines further integrated neoantigen prediction, treatment timing, combination strategies, manufacturing, and regulatory innovation (14). These analyses establish a general platform framework, but their application still depends on how differences in encoded function, manufacturing model, clinical treatment strategy, and regulatory classification affect the relevance of vaccine-derived evidence. Guidance from the United States Food and Drug Administration (FDA) for genome-editing products and draft guidance from the Medicines and Healthcare products Regulatory Agency (MHRA) for individualized cancer immunotherapies illustrate the differing scopes of relevant regulatory instruments (5, 9). Which elements of prophylactic-vaccine experience can support therapeutic development, and where are product-specific quality, nonclinical, and clinical data needed?

Drawing on illustrative development programs and regulatory instruments across five jurisdictions, this narrative review compares technical design, nonclinical and clinical evaluation, and regulatory classification to examine which evidence accumulated from prophylactic mRNA vaccines can support therapeutic development and where product-specific evidence remains necessary (**Figure 1**).

**Figure 1:**
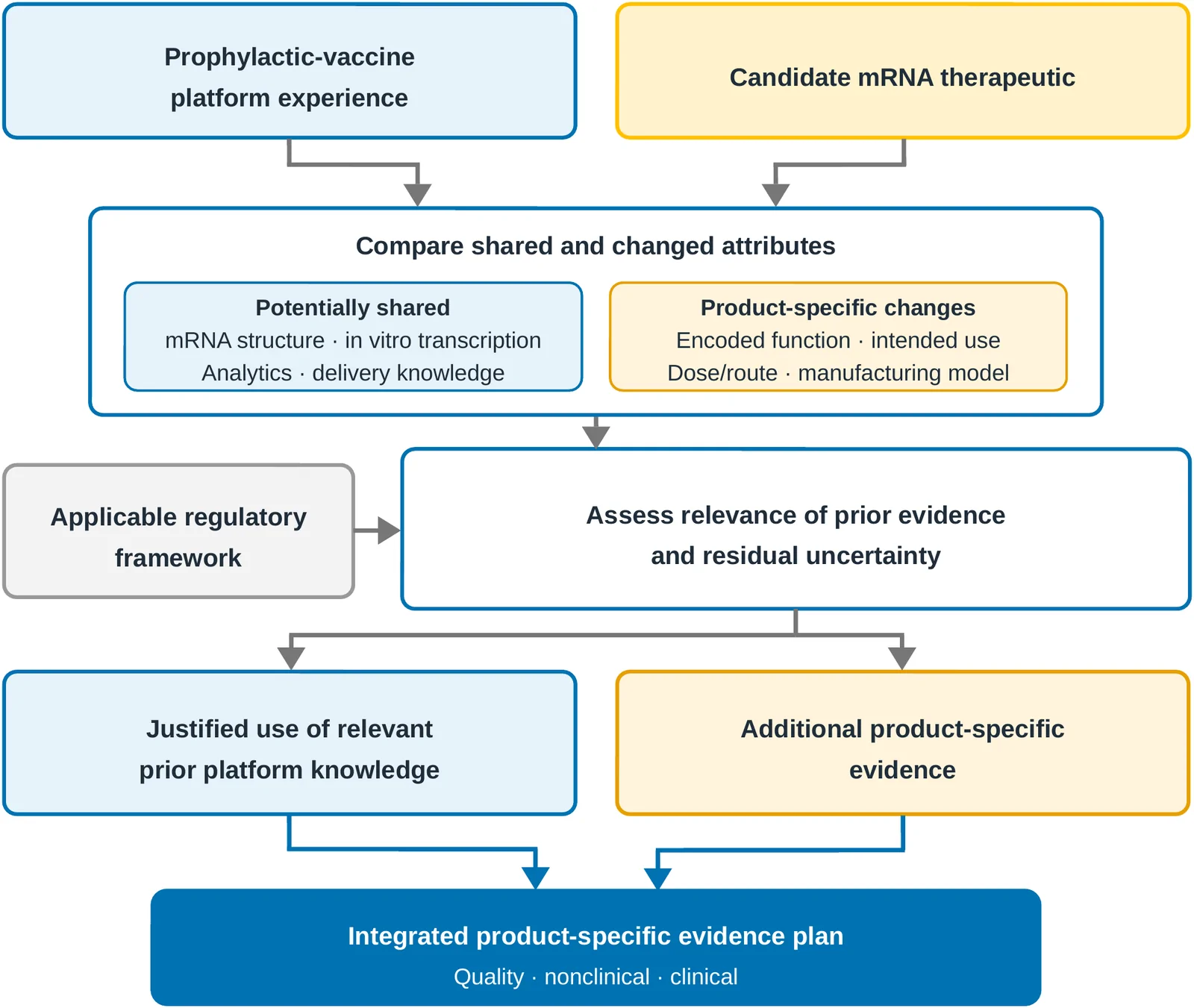
Framework for determining whether prophylactic-vaccine platform experience can support development of a candidate mRNA therapeutic. The ‘Compare shared and changed attributes’ step distinguishes potentially shared mRNA structure, *in vitro* transcription, analytics, and delivery knowledge from product-specific changes in encoded function, intended use, dose or route, and manufacturing model. Within the applicable regulatory framework, the ‘Assess relevance of prior evidence and residual uncertainty’ step determines whether relevant prior platform knowledge can be used with scientific justification and what additional product-specific evidence is required. These two components form the ‘Integrated product-specific evidence plan’ for quality, nonclinical, and clinical evaluation. Arrows indicate the sequence of assessment rather than automatic evidence transfer. mRNA, messenger RNA.

This narrative review used targeted searches of PubMed, ClinicalTrials.gov, and the official websites of FDA, the European Medicines Agency (EMA), MHRA, the Pharmaceuticals and Medical Devices Agency (PMDA), the Ministry of Food and Drug Safety (MFDS), the International Council for Harmonisation of Technical Requirements for Pharmaceuticals for Human Use (ICH), and the World Health Organization (WHO), covering sources available through October 13, 2026. Search concepts combined mRNA vaccines or therapeutics, individualized cancer immunotherapy, and platform technology with regulation, quality, manufacturing, nonclinical evaluation, and clinical development; product and trial names were also used. Sources were selected for direct relevance to platform expansion and the evaluation questions addressed here. Official regulatory documents were prioritized for regulatory statements and peer-reviewed primary studies for scientific and clinical claims; sponsor disclosures and registries were used for development status.

## 2 Technical comparison between mRNA vaccines and mRNA therapeutics

Beyond the core linear RNA architecture described above, conventional non-amplifying mRNA and saRNA medicinal products also share an *in vitro* transcription-based manufacturing process, delivery-system formulation, and control of common quality attributes (1, 6, 7, 15). Even on this shared platform, however, the principal design and evaluation considerations differ according to the nature of the expressed product and the intended pharmacological action, including the coding sequence, mechanism of action, role of the immune response, required expression profile, and manufacturing characteristics. Prophylactic mRNA vaccines encode pathogen-derived antigens and emphasize adequate immunogenicity and protective efficacy (16). Individualized vaccine-form mRNA cancer immunotherapies may encode multiple tumor neoantigens, making antigen breadth and T-cell priming central evaluation considerations (4, 9, 10). Non-vaccine mRNA therapeutics encode therapeutic proteins, cytokines, antibodies, or editing enzymes and therefore emphasize adequate expression in the target tissue, control of expression duration, suitability for repeat administration, and management of off-target risk where relevant (2, 3, 5). Immune responses also have contrasting roles: immune induction is central to the efficacy of prophylactic mRNA vaccines and vaccine-form therapeutics, whereas immune activation in a non-vaccine therapeutic may need to be minimized or precisely controlled according to the indication. From a manufacturing perspective, prophylactic mRNA vaccines are generally suited to standardized large-scale production, whereas vaccine-form therapeutics such as personalized mRNA cancer vaccines may require patient-specific or small-group production; non-vaccine therapeutics may use standardized or customized manufacturing models depending on the indication (**Table 1**).

**Table 1:** Technical comparison of prophylactic mRNA vaccines, vaccine-form mRNA therapeutics, and non-vaccine mRNA therapeutics.

Category	Prophylactic mRNA vaccine	Vaccine-form mRNA therapeutic	Non-vaccine mRNA therapeutic
Development objective	Prevent infectious disease and establish immune memory	Treat disease or reduce recurrence through antigen-specific immune modulation	Restore function, replace a protein, edit a gene, or modulate immunity
Representative example	Prophylactic mRNA vaccine	Personalized mRNA cancer therapy	Protein-replacement or gene-editing mRNA therapeutic
Expressed product	Pathogen-derived antigen	Treatment-directed antigen, including a neoantigen	Enzyme, therapeutic protein, antibody, cytokine, or editing enzyme
Basic mechanism of action	Antigen expression followed by humoral and cellular immune induction	Antigen expression followed by treatment-directed immune modulation	Expression of a therapeutic protein or editing enzyme
Role of the immune response	Central to efficacy	Central to efficacy	May need to be minimized or precisely controlled according to the indication
Required expression characteristics	Antigen expression sufficient to induce the intended immune response	Antigen expression and presentation sufficient for the intended immune response	Expression in the intended tissue or cellular compartment—or sufficient systemic exposure for a secreted product—with appropriate magnitude, biological activity, and duration
Manufacturing characteristics	Compatible with standardized large-scale production	Patient-specific batches for individualized products; standardized production for shared-antigen products	Standardized or indication-specific production
Principal development considerations	Immunogenicity, protective efficacy, and reactogenicity	Antigen selection, intended immune response, and product-specific treatment strategy	Biodistribution, repeat administration, functional testing, and off-target risk where relevant
Common technological foundation	mRNA design, *in vitro* transcription, purification, a product-appropriate delivery approach (including LNP formulation where applicable), and quality-attribute control		

Source: Authors’ synthesis from published reviews and regulatory guidance (1–6, 9, 15).

The term ‘vaccine-form mRNA therapeutic’ is descriptive and does not establish a legal classification.

LNP, lipid nanoparticle; mRNA, messenger RNA.

## 3 Research and development landscape of mRNA medicines

### 3.1 Expansion of authorized products and platform technologies

The authorized product landscape has expanded beyond the first coronavirus disease 2019 (COVID-19) mRNA vaccines to additional respiratory vaccines and an amplifying RNA platform. FDA approved mRESVIA in 2024 to prevent respiratory syncytial virus lower respiratory tract disease in adults aged 60 years and older and expanded the indication in 2025 to adults aged 18–59 years at increased risk (17, 18). FDA approved MNEXSPIKE in 2025 (19) and MFLUSIVA, an mRNA influenza vaccine, on August 5, 2026 for persons aged 50 years and older; the indication for persons aged 65 years and older was approved under the accelerated approval pathway (20). In the European Union, mCombriax was authorized in 2026 for active immunization against seasonal influenza and COVID-19 in adults aged 50 years and older, and KOSTAIVE received marketing authorization in 2025 as an saRNA COVID-19 vaccine (21, 22).

KOSTAIVE extends authorized RNA-vaccine technology beyond conventional non-amplifying mRNA. Its RNA encodes replicase proteins that generate additional intracellular copies of the antigen-encoding RNA (22–24). These authorizations demonstrate expansion across pathogen targets and RNA-amplification strategies, but they remain prophylactic products; transfer of their evidence to therapeutic applications still requires a product-specific scientific justification.

### 3.2 Clinical-development programs

Representative clinical-stage programs spanning vaccine-form therapeutics, protein and enzyme replacement, antigen-specific immune modulation, *in vivo* cell programming, and genome editing are summarized in **Table 2**. The table is deliberately selective rather than a market inventory and uses individual trial-registry records for row-level verification.

**Table 2:** Clinical-stage mRNA and related RNA therapeutic candidates.

Therapeutic class	Developer(s)	Candidate and encoded strategy	Indication	Highest publicly documented stage/status (trial identifier)
Vaccine-form, individualized	Moderna/Merck	Intismeran autogene (V940/mRNA-4157); linear mRNA encoding up to 34 patient-specific neoantigens	Melanoma and NSCLC	Phase 3; melanoma INTerpath-001 (NCT05933577): V940 plus pembrolizumab versus pembrolizumab alone met RFS and DMFS endpoints (27); separate NSCLC studies NCT06077760 and NCT06623422
Vaccine-form, individualized	BioNTech/Genentech	Autogene cevumeran (BNT122/RO7198457); linear mRNA encoding patient-specific neoantigens	Resected pancreatic cancer	Phase 2, active, not recruiting; NCT05968326
Vaccine-form, fixed antigen	BioNTech	BNT113; linear mRNA encoding HPV16 E6/E7 antigens	First-line unresectable recurrent/metastatic HPV16-positive, PD-L1-positive HNSCC	Phase 2/3, active, not recruiting; PFS dual primary endpoint not met for BNT113 plus pembrolizumab versus pembrolizumab alone; OS assessment ongoing; NCT04534205 (30)
Vaccine-form, saRNA	Immunomic Therapeutics	ITI-5000; saRNA encoding HERV-K and CT83 antigens fused to LAMP-1	Stage II–III triple-negative breast cancer	Phase 1, recruiting; NCT07652242
Vaccine-form, circRNA	Therorna	TI-0093; circRNA–LNP encoding HPV16 E6/E7 antigens	Recurrent or metastatic HPV16-positive solid tumors	Phase 1, recruiting; NCT07081984 (31)
Protein/enzyme replacement	Moderna/Recordati	mRNA-3927; two linear mRNAs co-formulated in LNPs, encoding the PCCA and PCCB subunits	Propionic acidemia	Phase 1/2 registrational study; target enrollment reached; NCT04159103 (32)
Protein/enzyme replacement	Arcturus Therapeutics	ARCT-810 (LUNAR-OTC); linear mRNA–LNP encoding OTC enzyme	Ornithine transcarbamylase deficiency	Phase 2; dosing completed and interim data reported; NCT06488313 (33)
Protein replacement, inhaled	Arcturus Therapeutics	ARCT-032; inhaled linear mRNA encoding CFTR	Cystic fibrosis	Phase 2, recruiting; NCT06747858 (34)
Vaccine-form, antigen-specific immunotherapy	Immunomic Therapeutics	ITI-9001; saRNA–LNP encoding CryJ2–LAMP-1	Japanese red cedar pollinosis	Phase 1, recruiting; NCT07726147
*In vivo* cell programming	AbbVie/Capstan Therapeutics	CPTX2309; targeted mRNA–LNP encoding an anti-CD19 CAR for transient programming of CD8-positive T cells	Rheumatoid arthritis and systemic lupus erythematosus	Phase 1, recruiting; NCT06917742
*In vivo* CRISPR–Cas9 editing	Intellia Therapeutics	Lonvoguran ziclumeran (lonvo-z/NTLA-2002); Cas9 mRNA + sgRNA LNP targeting KLKB1	Hereditary angioedema	Phase 3, active, not recruiting; endpoints met; BLA accepted with Priority Review; NCT06634420 (38, 39)
*In vivo* CRISPR–Cas9 editing	Intellia Therapeutics/Regeneron	Nexiguran ziclumeran (nex-z/NTLA-2001); Cas9 mRNA + sgRNA LNP targeting TTR	ATTR cardiomyopathy and hereditary ATTR polyneuropathy	Phase 3, recruiting after clinical holds were lifted; NCT06128629, NCT06672237 (38, 40)
*In vivo* base editing	Eli Lilly/Verve Therapeutics	VERVE-102 (LY4636581); adenine-base-editor mRNA + guide RNA in GalNAc–LNP targeting PCSK9	HeFH, premature CAD, or ASCVD risk	Phase 1b ongoing; Phase 2 registered, not yet recruiting; NCT06164730, NCT07839936 (41)
*In vivo* base editing	Beam Therapeutics	BEAM-302; liver-targeting LNP base-editing reagents designed to correct the SERPINA1 PiZ variant	Alpha-1 antitrypsin deficiency	Phase 1/2, recruiting; dosing underway in a pivotal cohort; NCT06389877 (42)

Source: Selected, non-exhaustive snapshot based on individual trial-registry records and sponsor disclosures checked through October 13, 2026. Each trial identifier links to its individual registry record.

Development stage/status does not imply regulatory approval or established clinical benefit. Circular RNA is included within the broader protein-coding RNA platform family but does not share the canonical linear mRNA architecture described in the text.

ASCVD, atherosclerotic cardiovascular disease; ATTR, transthyretin amyloidosis; BLA, biologics license application; CAD, coronary artery disease; CAR, chimeric antigen receptor; CD, cluster of differentiation; CFTR, cystic fibrosis transmembrane conductance regulator; circRNA, circular RNA; CRISPR–Cas9, clustered regularly interspaced short palindromic repeats–CRISPR-associated protein 9; DMFS, distant metastasis-free survival; CT83, cancer/testis antigen 83; CryJ2, major Japanese cedar pollen allergen Cry j 2; GalNAc, N-acetylgalactosamine; HeFH, heterozygous familial hypercholesterolemia; HERV-K, human endogenous retrovirus K; HNSCC, head and neck squamous cell carcinoma; HPV16, human papillomavirus type 16; LAMP-1, lysosome-associated membrane protein 1; KLKB1, kallikrein B1; LNP, lipid nanoparticle; mRNA, messenger RNA; NCT, ClinicalTrials.gov identifier; NSCLC, non-small cell lung cancer; OS, overall survival; OTC, ornithine transcarbamylase; PCCA, propionyl-CoA carboxylase subunit alpha; PCCB, propionyl-CoA carboxylase subunit beta; PCSK9, proprotein convertase subtilisin/kexin type 9; PD-L1, programmed death-ligand 1; PFS, progression-free survival; PiZ, proteinase inhibitor Z allele; RFS, recurrence-free survival; RNA, ribonucleic acid; saRNA, self-amplifying RNA; SERPINA1, serpin family A member 1; sgRNA, single-guide RNA; TTR, transthyretin.

Vaccine-form therapeutic development includes both individualized and fixed-antigen strategies. Intismeran autogene (mRNA-4157/V940) encodes up to 34 patient-specific neoantigens and is being evaluated in phase 3 melanoma and non-small cell lung cancer programs; the phase 3 INTerpath-001 trial of intismeran autogene plus pembrolizumab versus pembrolizumab alone met its recurrence-free-survival and distant-metastasis-free-survival endpoints at a prespecified interim analysis in August 2026 (25–27). BioNTech’s programs include the individualized neoantigen therapy autogene cevumeran and the fixed-antigen candidate BNT113 for human papillomavirus type 16 (HPV16)-positive head and neck cancer (28–30). BioNTech reported on October 13, 2026 that BNT113 plus pembrolizumab did not meet the progression-free-survival dual primary endpoint versus pembrolizumab alone; assessment of the overall-survival dual primary endpoint continues (30). Other vaccine-form candidates include the saRNA cancer vaccine candidate ITI-5000, the saRNA allergen immunotherapy ITI-9001, and TI-0093, a circRNA–LNP candidate in phase 1 development (31).

Non-vaccine programs broaden the development landscape beyond oncology. mRNA-3927 comprises two co-formulated mRNAs encoding the alpha and beta subunits of propionyl-CoA carboxylase (PCCA and PCCB, respectively) for propionic acidemia (32). ARCT-810 encodes ornithine transcarbamylase (OTC) for OTC deficiency; dosing in a phase 2 study was completed, and interim data were reported in September 2026 (33, 34). ARCT-032 is an inhaled mRNA candidate encoding cystic fibrosis transmembrane conductance regulator (CFTR) in phase 2 development for cystic fibrosis (34). Another early clinical example is CPTX2309, which uses targeted mRNA–LNP delivery to transiently program anti-CD19 chimeric antigen receptor (CAR) T cells *in vivo*; this mechanism raises evaluation questions distinct from those of prophylactic vaccination.

Delivery of editor-encoding mRNA together with a guide RNA enables transient editor expression, while the resulting edit may be durable (35–37). Lonvoguran ziclumeran targets the kallikrein B1 gene (KLKB1) for hereditary angioedema; its phase 3 trial met the primary and key secondary endpoints, and FDA accepted the biologics license application with Priority Review in September 2026 (38, 39). Nexiguran ziclumeran targets the transthyretin gene (TTR) in transthyretin amyloidosis, with phase 3 development continuing after clinical holds were lifted (38, 40). Base-editing programs targeting proprotein convertase subtilisin/kexin type 9 (PCSK9) and serpin family A member 1 (SERPINA1) further extend the range of mRNA-delivered editors in clinical development (41, 42).

## 4 Comparison of evaluation considerations for prophylactic mRNA vaccines and mRNA therapeutics

For prophylactic mRNA vaccines, induction of an antigen-specific immune response is a principal component of efficacy (16, 43). Most non-vaccine mRNA therapeutics instead aim to express a defined functional protein, and nonspecific immune activation may represent reactogenicity, toxicity, or another adverse effect that should be minimized (1, 44). For vaccine-form cancer therapeutics, tumor-antigen-specific immunity is intended, although nonspecific systemic inflammation remains undesirable (4, 10, 45). The focus of evaluation therefore depends on whether a particular immune response constitutes pharmacological activity or a safety risk, while all mRNA–delivery-system products require systematic assessment of toxicities arising from the RNA, delivery system, expressed product, and their interactions.

### 4.1 Biodistribution and persistence

Biodistribution and persistence are central nonclinical considerations for mRNA–delivery-system products because distribution of the administered mRNA and the delivery system—including the LNP and relevant lipid components—beyond the administration site, their accumulation or retention in particular tissues, and their persistence may be related to systemic toxicity and potential immunotoxicity, including inflammation or hypersensitivity. A fit-for-purpose assessment should characterize the presence, persistence, and clearance of the administered mRNA; retention and potential accumulation of the LNP and relevant lipid components; and distribution across a scientifically justified panel that includes target and non-target organs, the administration site, draining lymph nodes, and relevant biofluids (11, 16, 46, 47).

Approved prophylactic mRNA vaccines are generally administered intramuscularly (16), whereas experimental mRNA–LNP formulations have also been evaluated by subcutaneous and intradermal routes in mice (48). For these products, conventional plasma pharmacokinetic or absorption, distribution, metabolism, and excretion studies are normally not required and may be less informative than a fit-for-purpose, tissue-based assessment of unintended distribution to non-target organs (16, 49, 50). Local administration should not, however, be equated with confinement to the injection site (50). If a change to the mRNA construct or formulation increases stability or prolongs expression, the sampling period should be sufficient to characterize persistence and clearance in non-target tissues and any related toxicological risk (11, 47). EMA’s assessment of mRNA-1273 provides a product-specific precedent: rather than conducting a dedicated study with mRNA-1273, biodistribution was evaluated in a non-good-laboratory-practice, single-dose intramuscular rat study of mRNA-1647 formulated in the same SM-102-containing LNP; EMA also indicated that a new formulation or excipient may require dedicated distribution studies (50). This precedent is not a general waiver and depends on the relevance of the bridging justification.

When an mRNA therapeutic is classified as a gene-therapy product or evaluated under gene-therapy principles, biodistribution and persistence expectations are generally more explicit and product-specific. Adequate exposure in the intended target tissue may be integral to efficacy, and repeated administration may require assessment of accumulation of the RNA, delivery-system components, expressed product, or downstream biological effects (11, 44, 46, 47, 51). Study design should be determined by the intended tissue, route, dose, interval, expression duration, delivery-system properties, and overall risk profile rather than by the mRNA platform label alone (11, 52). Conventional mRNA acts in the cytoplasm and is not intended to integrate into chromosomes, but this characteristic does not by itself waive the need to evaluate biodistribution (6, 16, 44). When the manufacturing process, composition, delivery system, route, dose, and expression profile are sufficiently similar to those of an authorized product, relevant prior platform data may support a scientifically justified reduction or modification of a new biodistribution program (46, 47, 50). Whether a separate study may be reduced or omitted should be discussed prospectively with the relevant regulatory authority.

### 4.2 Pharmacological efficacy evaluation

Even when the same mRNA–delivery-system platform is used, pharmacological efficacy endpoints and their interpretation differ according to the development objective—prevention or treatment—and the form of expected clinical benefit (1, 16, 44). For prophylactic mRNA vaccines, evaluation focuses on the presence, magnitude, quality, and durability of humoral and cellular immune responses relevant to infection prevention and, when necessary, their relationship to protection. Nonclinical programs therefore commonly measure antigen-specific binding and neutralizing antibodies, T-cell responses, and T-helper 1/T-helper 2 polarization; when a suitable challenge model exists, reduced pathogen replication, disease severity, or pathology may be used to demonstrate protection (16, 49). For tissue-targeted protein-replacement or gene-therapy-like mRNA products, evaluation should demonstrate delivery to the intended tissue, expression of a correctly processed and functional protein at the intended level, and improvement in a disease-relevant physiological measure, biomarker, or phenotype (44, 51, 52). Demonstrating the *in vivo* mechanism of such products requires a biologically relevant species or model in which the introduced sequence can reach the target, be expressed at the expected level, and generate the intended functional activity; if quality data indicate the possibility of an aberrant gene product, its potential biological consequences should also be evaluated (44, 52). For products encoding a secreted protein, circulating exposure and the intended downstream functional response may be more appropriate pharmacodynamic measures, while unwanted immune activation should be monitored and minimized (2, 3). For vaccine-form cancer therapeutics, antigen-specific T-cell responses and tumor effects provide pharmacodynamic and efficacy measures, respectively (53). **Table 3** summarizes illustrative efficacy and pharmacodynamic endpoints by therapeutic application.

**Table 3:** Efficacy and pharmacodynamic endpoints for selected mRNA therapeutic applications.

Illustrative application	Efficacy endpoints	Mechanistic and pharmacodynamic endpoints	Illustrative methods	Refs.
Protein replacement or expression (e.g., enzyme, hormone, growth factor)	Functional recovery in a disease model, including survival, symptom score, functional testing, or histopathology	Target-tissue protein concentration and duration; downstream pathway biomarker	ELISA, LC–MS/MS, immunohistochemistry, western blotting, enzyme-activity assay, physiological testing, or imaging	(1–3, 85)
Enzyme replacement in metabolic disease	Improvement in a disease-relevant function or phenotype	Increased enzyme activity and reduction or normalization of a pathway-specific toxic metabolite	Plasma or tissue metabolite analysis by LC–MS, enzyme-activity assay, and histopathology	(51, 85, 86)
mRNA-expressed antibody	Disease protection or improvement in a disease-relevant outcome after antibody expression	*In vivo* concentration or exposure of the expressed antibody; target binding, neutralization, or functional blockade	Antibody quantification by ELISA, neutralization or functional assay, and receptor-binding or blocking assay	(87)
Expression of an immunomodulatory protein (e.g., cytokine)	Tumor-growth inhibition or prolonged survival	Immune-cell activation or infiltration and cytokine profile, interpreted together with the efficacy–toxicity window	Flow cytometry, intracellular cytokine staining, ELISpot, tissue immunostaining, tumor volume, or survival	(4, 88)
Therapeutic mRNA cancer vaccine (antigen expression and T-cell induction)	Tumor-growth inhibition or survival in a tumor model and, where appropriate, protection after rechallenge	Antigen-specific CD8+ and CD4+ responses, tumor-infiltrating lymphocytes, and cytotoxic function	ELISpot, intracellular cytokine staining, MHC multimer assay, intratumoral immune-cell analysis, tumor volume, survival, or neoantigen-specific immunogenicity	(4, 10, 53)
Genome editing	Improvement of a disease phenotype after restoration or modification of target-gene function	On-target editing rate, the intended change in the target gene product—including restoration, reduction, or altered function—and a downstream pathway biomarker	Next-generation or amplicon sequencing, protein quantification, and functional testing	(5, 35, 37, 89, 90)

Source: illustrative authors’ synthesis.

Rows are illustrative and may overlap; they do not define separate regulatory product categories. The appropriate endpoint and assay set is product- and mechanism-specific. Off-target editing and genomic-integrity assessments are safety-characterization endpoints rather than primary pharmacodynamic endpoints (5, 44).

CD, cluster of differentiation; ELISA, enzyme-linked immunosorbent assay; ELISpot, enzyme-linked immunospot; LC–MS(/MS), liquid chromatography–mass spectrometry (or tandem mass spectrometry); MHC, major histocompatibility complex; mRNA, messenger RNA.

### 4.3 Toxicity assessment

Because prophylactic mRNA vaccines are generally administered intramuscularly, safety evaluation typically emphasizes the injection site, draining lymph nodes, relevant immune organs, and systemic findings associated with local tolerance and repeat administration. Their clinical schedules are usually limited in dose number and duration, and many changes observed in repeat-dose studies fall within an anticipated range of local or immune responses (16, 43, 49). Therapeutic products may instead be administered intravenously or by an organ-directed route—including hepatic, ocular, or intrathecal administration—and may require chronic or intermittent repeat dosing. Their toxicity programs may therefore need to address route- and organ-specific findings, cumulative toxicity, innate and adaptive immune activation, antidrug or neutralizing antibodies to the encoded protein or another relevant component, and the reversibility or persistence of findings in recovery groups (44, 52).

Toxicity studies should use a pharmacologically relevant species where possible and consider the animals’ physiological state, the dose, route, method, frequency, and duration of administration, and the activity of the expressed product (44, 52). If no conventional species is responsive, a human-receptor transgenic, genetically modified, humanized, or disease model, or a homologous surrogate protein or construct, may be appropriate when its relevance and limitations are justified. Talimogene laherparepvec (Imlygic), an oncolytic herpes simplex virus type 1 gene-therapy product rather than an mRNA product, provides an instructive analogy: because human granulocyte-macrophage colony-stimulating factor has limited biological activity in mice, mouse studies used a surrogate virus encoding murine granulocyte-macrophage colony-stimulating factor (54). Routine genotoxicity or carcinogenicity studies are rarely required for conventional prophylactic mRNA vaccines unless a novel component or other product-specific concern creates a plausible risk (16, 49). For a therapeutic product, the biological activity and duration of the expressed product, non-target distribution, chronic inflammation, and any editing or genomic-integrity risk should be considered according to its risk profile (5, 44, 47); for an expressed protein with plausible tumorigenic potential, a weight-of-evidence approach to carcinogenicity follows general biopharmaceutical principles (55).

## 5 Regulatory frameworks for therapeutic mRNA products and future outlook

Regulatory science for mRNA platforms is expanding from experience with the quality, nonclinical, and clinical evaluation of prophylactic mRNA vaccines against COVID-19 to individualized cancer immunotherapy, rare-disease therapeutics, delivery of gene-editing enzymes, and sequence design supported by artificial intelligence or other algorithms (5, 9). Compared with the established regulatory experience for prophylactic mRNA vaccines, regulatory approaches for therapeutic mRNA products remain less standardized and more dependent on the product’s intended use, mechanism of action, and jurisdiction-specific classification (56, 57).

Regulatory approaches are nevertheless becoming more specific. MHRA’s 2025 draft guideline for individualized mRNA cancer immunotherapies provides a concrete setting in which to distinguish shared platform controls from patient-specific evidence; this product class is therefore examined in greater depth below (9). Because the draft and consultation response do not establish binding final requirements, they are interpreted here as an emerging regulatory direction (58).

### 5.1 Regulatory classification of prophylactic mRNA vaccines and mRNA therapeutics across jurisdictions

Across the jurisdictions reviewed, classification turns principally on intended use and mechanism of action rather than on the mere use of an mRNA platform. An mRNA product that expresses an antigen to prevent an infectious disease is generally regulated through a vaccine or biological-product pathway, whereas an mRNA product intended to treat disease, restore function, induce tumor-antigen-specific therapeutic immunity, or express a gene-editing enzyme may be considered within gene-therapy medicinal product (GTMP), advanced therapy medicinal product (ATMP), therapeutic cancer-vaccine, or other medicinal-product frameworks, depending on the jurisdiction and product (9, 45, 57, 59–62).

In the United States, FDA-approved prophylactic mRNA vaccines such as mRESVIA, MNEXSPIKE, and MFLUSIVA are licensed under biologics license applications and reviewed within FDA’s Center for Biologics Evaluation and Research (17, 19, 20, 56). An mRNA therapeutic should not, however, be assumed to follow an identical review route solely because it uses mRNA. FDA Center assignment and applicable guidance depend on the particular product: vaccines and gene-therapy products remain within the Center for Biologics Evaluation and Research, while categories of therapeutic proteins identified in FDA’s transfer policy fall within the Center for Drug Evaluation and Research (59, 63). For combination products, Center assignment is determined according to the product’s primary mode of action (63).

FDA’s Long Term Follow-Up After Administration of Human Gene Therapy Products: Guidance for Industry includes products containing genetically modified cells and gene-therapy products whose effect is mediated by transcription or translation of transferred genetic material, including *in vitro*-transcribed RNA, while excluding vaccines against infectious diseases (59). FDA’s Clinical Considerations for Therapeutic Cancer Vaccines: Guidance for Industry likewise states that vaccines intended to prevent infectious diseases are outside its scope (45). These distinctions show that a vaccine-like formulation intended to treat cancer is evaluated under considerations that differ from those for a prophylactic mRNA vaccine.

For mRNA-delivered genome-editing products, FDA’s April 2026 draft guidance provides recommendations for next-generation sequencing (NGS)- and bioinformatics-based nonclinical assessment of on-target and off-target editing and chromosomal integrity. The draft supplements the January 2024 final guidance and discusses *in vivo* editing with LNP-delivered editor mRNA; its recommendations concern genome-editing products, not mRNA therapeutics in general (5, 64).

EMA’s draft Guideline on the Quality Aspects of mRNA Vaccines addresses mRNA vaccines against infectious diseases, including non-replicating and self-amplifying RNA, while placing other mRNA-based medicinal products outside its scope, although relevant principles may be applicable to them (15).

Under the current European Union framework, vaccines against infectious diseases are excluded from the GTMP definition. Published Committee for Advanced Therapies (CAT) recommendations nevertheless provide direct therapeutic-mRNA precedents: patient-specific mRNA encoding tumor-derived neoepitopes, multiple *in vitro*-transcribed mRNAs encoding tumor antigens or immunomodulatory cytokines, and mRNAs encoding CFTR or ornithine transcarbamylase for protein replacement have been classified as GTMPs (57, 60). These are product-specific scientific recommendations rather than an automatic classification of all therapeutic mRNA products; when classification remains uncertain, developers may request an Article 17 ATMP-classification recommendation (65).

The Council’s September 2026 first-reading position on the revised pharmaceutical Directive would define GTMPs to include sequence-specific genome-editing products and recombinant or synthetic nucleic acids whose effects are mediated by long-lasting transcription or translation, while retaining the exclusion for vaccines against infectious diseases. Because the text had not entered into force at the search cutoff, it is treated here as a pending legislative development rather than current European Union law (66).

Under regulation 2A of the Human Medicines Regulations 2012, a GTMP contains or consists of a recombinant nucleic acid and vaccines against infectious diseases are excluded from the GTMP definition. MHRA’s draft Guideline on Individualised mRNA Cancer Immunotherapies reports that products within its scope are currently classified as ATMPs, subclassified as gene therapies, while advising that classification be agreed case by case (9, 67).

A separate MHRA consultation published in May 2026 proposed modernizing the statutory GTMP definition by removing the biological-origin limitation and expressly covering recombinant or synthetic nucleic acids intended to regulate, replace, or add a nucleic-acid sequence and whose effect is mediated by transcription or translation, together with sequence-specific genome-editing products irrespective of the nature of the active substance. The proposal would retain the exclusion for vaccines against infectious diseases. Unlike the Council’s European Union position, the proposed United Kingdom definition does not contain a ‘long-lasting’ transcription-or-translation qualifier and expressly considers transiently expressed mRNA immunotherapies. At the search cutoff, the consultation had closed but the proposed definition had not been enacted (68).

Japan also distinguishes these categories in legislation and regulatory organization. Under the Pharmaceuticals and Medical Devices Act, PMDA lists gene therapy among regenerative medical products but expressly excludes prophylactic vaccines against infectious diseases; prophylactic mRNA vaccines are therefore reviewed as pharmaceuticals. PMDA provides separate review information for vaccines and blood products and for regenerative medical products, reflecting distinct regulatory frameworks for these product groups (61, 69).

In the Republic of Korea, MFDS has issued separate guidance for prophylactic mRNA vaccines and mRNA-based gene-therapy products, as well as dedicated guidance on personalized neoantigen-targeted therapies, gene-therapy products that use genome editing, potency-assay development, and comparability assessment of investigational cell and gene therapy products. The personalized-product guidance covers DNA-, mRNA-, and dendritic-cell-based products regulated as advanced biological products and addresses tumor and normal-tissue sequencing, neoantigen selection, patient-specific manufacture, and quality, nonclinical, and clinical evaluation (43, 46, 52, 70–74). These documents are non-binding guidance and do not establish automatic acceptance of platform data; their applicability remains product specific. **Table 4** compares selected instruments and classification precedents across jurisdictions.

**Table 4:** mRNA regulatory instruments and classification precedents across jurisdictions.

Jurisdiction/authority	Current classification or precedent	Relevant instrument and status	Scope and limits
United States (FDA)	Prophylactic mRNA vaccines are biological products; therapeutic mRNA is evaluated according to intended use and may be subject to therapeutic-cancer-vaccine, therapeutic-biologic, or gene-therapy considerations (45, 56, 59, 63).	Therapeutic-cancer-vaccine, human genome-editing, and gene-therapy follow-up guidances [Final]; NGS genome-editing safety, active-immunotherapy potency, prior-knowledge genome-editing, and platform-designation guidances [Draft] (5, 45, 59, 64, 75, 82, 84).	The active-immunotherapy draft excludes preventive infectious-disease vaccines. The NGS draft concerns genome-editing products, including mRNA-delivered editors, rather than mRNA products generally; platform designation is not approval and long-term follow-up remains risk based (59, 64, 75, 82).
European Union (EMA/CAT)	Vaccines against infectious diseases are excluded from the current GTMP definition. CAT precedents classify patient-specific neoepitope, multi-antigen or immunomodulatory, and CFTR or OTC protein-replacement mRNA products as GTMPs (57, 60, 65).	Current GTMP definition [Law]; GTMP guideline [Final]; mRNA-vaccine quality guideline [Draft]; CAT mRNA classifications [Scientific recommendation]; revised pharmaceutical Directive [Council first-reading position] (not in force) (15, 44, 57, 60, 65, 66).	CAT recommendations are product specific. The Council position would revise the GTMP definition and establish a certified platform technology master file applicable to mRNA, but reliance would require evidence that common data remain applicable and the text was not in force at the cutoff (66).
United Kingdom (MHRA)	Current law excludes vaccines against infectious diseases from GTMPs; the 2025 draft reports individualized mRNA cancer immunotherapies as ATMPs/GTMPs, subject to case-by-case agreement (9, 67).	Human Medicines Regulations 2012, regulation 2A [Law]; individualised mRNA cancer-immunotherapy guideline [Draft] and response [Consultation response]; GTMP-definition proposal [Closed consultation] (not law) (9, 58, 67, 68).	The 2026 proposal would cover recombinant or synthetic nucleic acids intended to regulate, replace, or add a nucleic-acid sequence and acting through transcription or translation, as well as sequence-specific genome editing, without a duration qualifier. No amended definition had been adopted at the cutoff (68).
Japan (PMDA)	Prophylactic mRNA vaccines are distinguished from regenerative medical products, which include gene-therapy products (61, 69).	Regenerative-product framework [Established]; mRNA–LNP vaccine platform notice [Early consideration] (61, 83).	The platform notice concerns nonclinical safety of prophylactic mRNA vaccines, not therapeutic mRNA or its follow-up requirements (83).
Republic of Korea (MFDS)	Separate guidance addresses prophylactic mRNA vaccines, mRNA-based gene-therapy products, genome-editing gene-therapy products, personalized neoantigen-targeted therapies using DNA, mRNA, or dendritic cells, potency assays, and comparability of investigational cell and gene therapy products (43, 70–74).	mRNA-vaccine, mRNA-gene-therapy quality, personalized neoantigen-targeted therapy, genome-editing quality/nonclinical/clinical, cell- and gene-therapy potency, gene-therapy nonclinical/biodistribution, and investigational-product comparability guidances [Final] (43, 46, 52, 70–74).	The personalized, genome-editing, potency, and comparability guidances address patient-specific development, editing-specific evaluation, assay development and validation, and manufacturing-change comparability, respectively, but are nonbinding and do not create automatic platform-data reuse (71–74).

Selected, non-exhaustive comparison; status checked October 13, 2026. [Law] is binding; [Established], an existing framework; [Final], adopted nonbinding guidance; [Draft], proposed guidance; [Consultation response], an agency response; [Early consideration], preliminary principles; and [Scientific recommendation], product-specific CAT advice. [Council first-reading position] and [Closed consultation] were not in force. Classification and platform-data acceptance remain product and jurisdiction specific.

ATMP, advanced therapy medicinal product; CAT, Committee for Advanced Therapies; CFTR, cystic fibrosis transmembrane conductance regulator; DNA, deoxyribonucleic acid; EMA, European Medicines Agency; FDA, United States Food and Drug Administration; GTMP, gene-therapy medicinal product; LNP, lipid nanoparticle; MFDS, Ministry of Food and Drug Safety; MHRA, Medicines and Healthcare products Regulatory Agency; mRNA, messenger RNA; NGS, next-generation sequencing; OTC, ornithine transcarbamylase; PMDA, Pharmaceuticals and Medical Devices Agency; RNA, ribonucleic acid.

### 5.2 Patient-specific design, manufacturing, and control of individualized mRNA cancer immunotherapies

Unlike a prophylactic mRNA vaccine with a fixed sequence, an individualized mRNA cancer immunotherapy may contain a different mRNA sequence and antigen combination for each patient according to the molecular characteristics of that patient’s tumor. MHRA describes products tailored to an individual tumor and notes that a deterministic algorithm or artificial intelligence may be used to design a treatment matching patient-specific tumor characteristics. The MHRA draft therefore frames assessment of these products as end-to-end management linking patient input data, algorithmic design, manufacture, release, distribution, and administration (9, 58).

A central issue is that artificial intelligence, machine learning, and bioinformatics can move beyond research support and directly influence the design and quality of the finished medicinal product (9, 58). Stakeholder feedback on the MHRA draft raised questions about applying good manufacturing practice (GMP) change control to predictive models used for manufacture, risk-based management of model updates, software-version records, and patient-level manufacturing traceability. Proposals to distinguish deterministic models from adaptive or continuously learning models appeared in consultation feedback and should be treated as issues for further regulatory development rather than as final binding requirements.

Patient-specific release testing. Individualized mRNA therapeutics require a fit-for-purpose interpretation of the traditional batch concept. The MHRA draft proposes patient-batch controls for mRNA identity, concentration and purity, delivery-system attributes such as particle size and encapsulation efficiency, and sterility and endotoxin. The location and timing of individual tests should be justified within the control strategy; validated in-process controls may support omission of selected finished-product tests, but do not eliminate patient-batch identity or traceability. Rapid microbial methods or two-stage testing are discussed as case-specific options requiring supporting data and a justified benefit–risk assessment, not routine exemptions from quality assurance (9, 58).

Representative-batch selection. Full stability studies on every patient-specific batch may consume limited material and be impracticable within treatment timelines. Earlier platform analyses proposed several ways to characterize patient-specific products: comparing batches made from a common DNA template in parallel manufacturing streams, evaluating designed sequences that span the anticipated range of patient-specific variation, and assessing changes to genomic sequencing or bioinformatics at the level of the overall process (12). A later lifecycle review similarly proposed representative batches spanning the expected range and boundaries of mRNA length and structure (13). The MHRA consultation response likewise supports representative batches covering anticipated sequence diversity, length, and structure (58). Together, these approaches support selecting batches that bracket the relevant sequence range and challenge manufacturing performance, formulation, and stability. Such shared evidence can support process knowledge and shelf-life justification, while patient-specific release controls remain necessary.

Potency assessment. The MHRA draft distinguishes protein-expression functionality assays from a true measure of biological potency. Expression testing can support a control strategy, but any use as a correlate of the intended biological effect requires supporting data. The consultation response acknowledges that separate potency assays for every unique neoantigen ensemble are impracticable, while retaining an expectation for an appropriate *in vitro* assay linked to biological effect. Representative constructs can help investigate this link during development, but an expression result alone should not be interpreted as evidence of clinical benefit (9, 58). FDA’s August 2026 draft guidance addresses active immunotherapy products. For personalized vectored products, including RNA vectors, it recommends a bioassay for vector-related function, while allowing the activity of patient-specific epitopes to be supported by a qualified manufacturing process and bioinformatics pipeline together with sequence confirmation and other physicochemical testing; a separate patient-specific epitope bioassay for every lot may therefore be unnecessary (75).

Algorithm and process changes. The MHRA draft proposes keeping product-design steps fixed throughout pivotal trials and justifying whether subsequent changes preserve the relevance of the clinical evidence. For practical change management, we suggest recording the algorithm version used for each patient, assessing effects on antigen selection and product quality, and using risk-proportionate comparability evidence for algorithm or manufacturing changes. GMP oversight of manufacture-related design is supported in the consultation response, but detailed change-management approaches remain under regulatory development (9, 58).

Another emerging boundary is whether performance of an artificial-intelligence or algorithmic component should be assessed as a separate software product or controlled under pharmaceutical GMP as part of manufacture (9, 58). MHRA’s consultation response states that the agency supports GMP oversight when neoantigen selection is used in product manufacture and will consider this approach in the final guideline; medical-device or *in vitro* diagnostic requirements may apply when software has an independent medical purpose and is not used in manufacture. The applicable boundary remains dependent on the intended purpose and implementation of the software.

### 5.3 Clinical evaluation of individualized mRNA cancer immunotherapies

Patient-specific sequences do not inherently preclude randomized trials: participants, rather than sequences, are randomized, and the intervention can be defined prospectively as a treatment strategy governed by common antigen-selection, manufacturing, and release controls. The randomized KEYNOTE-942 study provides a clinical example (76).

#### 5.3.1 Combination therapies

For products administered with immune-checkpoint inhibitors or other anticancer therapy, an add-on comparison using the same background regimen in both groups can estimate the incremental benefit of adding the individualized immunotherapy. KEYNOTE-942 compared mRNA-4157 plus pembrolizumab with pembrolizumab alone (76). Such a comparison, however, does not establish the efficacy of the immunotherapy as monotherapy. Concomitant treatment, dose, schedule, and sequencing should be justified (9, 45), and the protocol should specify how immune-mediated adverse events common to both treatments will be attributed.

#### 5.3.2 Clinically meaningful endpoints

Endpoint selection should reflect disease stage and the intended clinical benefit. In the adjuvant setting, recurrence-free survival and distant-metastasis-free survival can evaluate recurrence prevention; KEYNOTE-942 used recurrence-free survival as its primary endpoint (76). Overall survival remains important but may require longer follow-up and is influenced by subsequent therapy. Neoantigen-specific T-cell responses, including enzyme-linked immunospot (ELISpot) results, support pharmacodynamic and exploratory evaluation but should not be treated as validated surrogates for clinical benefit. FDA’s therapeutic cancer-vaccine guidance also notes that delayed treatment effects may violate proportional-hazards assumptions; follow-up duration, trial maturity, and the analysis method should therefore be considered prospectively (45).

#### 5.3.3 Manufacturing delays

Tumor sampling, sequencing, antigen selection, manufacture, and release introduce a patient-specific interval before first dose. In the pancreatic-cancer study of autogene cevumeran, the prespecified benchmarks were completion of manufacture within six weeks and first administration within nine weeks after surgery (77). These are study-specific benchmarks, not universal turnaround times. Recurrence, deterioration, or manufacturing failure during this interval can prevent dosing. Trials should therefore report the tissue-to-dose interval, production success, reasons for non-administration, and interim treatment. The protocol should specify whether background therapy starts during manufacture, rather than assume all treatment awaits product availability (9, 45).

#### 5.3.4 Analysis populations

The timing of randomization relative to manufacture determines the population and treatment strategy evaluated. Randomization before manufacture allows the assigned-strategy comparison to include manufacturing failure, delay, and non-initiation; randomization only after successful manufacture limits inference to patients with an available product who remain eligible (9). An intention-to-treat analysis should include randomized participants according to assigned strategy, including those who do not receive the manufactured product. Restricting efficacy analyses to patients who received the product can select those who remained eligible through the waiting period and exclude early events; requiring survival until vaccination can produce guarantee-time or immortal-time bias (78). Using ICH E9(R1), the protocol should prespecify the treatment conditions, population, endpoint, summary measure, and handling of treatment changes or non-initiation (79). Manufacturing failure, delay, or non-initiation before dosing should be handled as prespecified for the assigned treatment strategy. Recurrence or death included in the endpoint should be counted as endpoint events.

### 5.4 Expanding the use of limited patient data and mechanism-based evidence in rare genetic diseases

As mRNA therapeutics expand into rare diseases and ultra-rare variants, there may be more situations in which a traditional large randomized trial is not feasible because the eligible population is intrinsically small. For nucleic-acid or gene-based treatments targeting such conditions, repeatedly administering an identical product to a sufficiently large population may be difficult. Regulatory approaches for intrinsically small populations therefore consider how limited patient data can be interpreted together with mechanistic plausibility, biomarkers, natural-history information, external controls, pharmacodynamic data, nonclinical evidence, and chemistry, manufacturing, and controls information (80, 81).

In February 2026, FDA issued the draft Plausible Mechanism Framework for individualized therapies targeting specific severe or life-threatening genetic conditions with a known biological cause and very few available patients (80). The draft emphasizes that clinical observations must be evaluated with nonclinical and chemistry, manufacturing, and controls evidence and that the product must be manufactured to appropriate quality standards. It describes how one adequate and well-controlled investigation together with confirmatory evidence may support effectiveness when patient availability is extremely limited, using the untreated disease course, the plausibility of the treatment mechanism, target engagement or correction, clinically meaningful change, and biomarkers as relevant components of the evidence.

The Plausible Mechanism Framework is not a separate approval pathway for all mRNA therapeutics, nor is it directed specifically to individualized cancer immunotherapy. Its concepts may be relevant to an individualized RNA-based or genome-editing therapy only when the product and disease meet the framework’s narrow scope (80). This small-population rationale should not be extended to individualized cancer immunotherapy merely because patients receive different sequences; randomized strategy comparisons remain possible (76). Even when clinical evidence is necessarily limited, safety, quality, manufacturing control, and other statutory requirements remain applicable.

FDA’s 2025 draft guidance Innovative Designs for Clinical Trials of Cellular and Gene Therapy Products in Small Populations reflects the same broader direction. It discusses approaches such as single-arm designs, use of participants as their own controls, disease-progression models, externally controlled trials, adaptive and Bayesian methods, and master protocols for cellular and gene-therapy products in small populations. These options are program-specific and become relevant to an mRNA therapeutic only if the product falls within the scope of the guidance (81).

This use of diverse evidence does not lower the evidentiary standard; it broadens the types of evidence and methods of interpretation available when patient numbers are intrinsically limited (80, 81). Developers should therefore plan early for natural-history data, disease-specific biomarkers, pharmacological effects of the expressed product, within-patient baseline trajectory, functional outcomes after treatment, and long-term safety monitoring. For an mRNA-delivered genome editor, transient mRNA expression does not imply transient biological risk because the edit may persist; mechanistic evidence should therefore be integrated with follow-up appropriate to the durability and delayed-risk profile of the edit (5, 59).

### 5.5 Importance of early regulatory consultation and regulatory information tracking amid limited public precedent

Because classification and evidence requirements for therapeutic mRNA products remain product- and jurisdiction-specific, developers should engage the relevant regulatory authority early in development. Early consultation can clarify the applicable regulatory pathway, nonclinical and clinical evidence expectations, clinical endpoints, and postauthorization plans before the submission dossier is finalized (9, 57, 80, 81).

Individualized mRNA cancer immunotherapies combine characteristics of prophylactic mRNA vaccines, anticancer medicines, gene therapies, ATMPs, and software- or algorithm-linked manufacturing. EMA/CAT classification precedents, MHRA’s draft individualized mRNA cancer-immunotherapy guidance, and FDA’s therapeutic cancer-vaccine guidance collectively show why existing labels alone may not adequately define the evidence package (9, 45, 57). Early consultation can clarify regulatory identity, the unit of authorization, the scope of reusable platform evidence, and the boundaries of product-specific risk assessment.

During early consultation, developers should distinguish fixed platform elements—such as the mRNA backbone, delivery-system composition, manufacturing operations, and analytical methods—from elements that vary by patient, including input data, coding sequence, antigen combination, or guide sequence. Platform knowledge may be more transferable for a prophylactic mRNA vaccine, whereas evidence requirements for a therapeutic product can change with the function of the expressed product, target-tissue delivery, repeat administration, off-target risk, or patient-specific sequence variation. The central question should therefore be which data are scientifically common to the platform and which risks remain patient- or product-specific (9, 58, 82–84).

### 5.6 Parallel use of platform common data and product-specific risk assessment

Earlier reviews and the regulatory instruments examined here support use of scientifically justified common platform knowledge while retaining product-specific assessment (9, 12, 13, 15, 58, 82–84). For prophylactic mRNA vaccines, a platform-based approach may leverage an unchanged LNP composition, mRNA backbone, manufacturing process, analytical procedures, and impurity profile, whereas a new antigen, saRNA amplification mechanism, or antigen ratio in a multivalent vaccine remains product-specific. For individualized mRNA cancer immunotherapies, patient-specific sequence variation, antigen selection, manufacturing traceability, and the function of the expressed product require product-specific assessment even when common platform data can be leveraged. For protein-replacement mRNA therapeutics, common process and analytical knowledge may support manufacturing evaluation, whereas target-tissue delivery, the level and duration of functional protein expression, and safety under the intended repeat-dose regimen remain product-specific (2, 3, 12, 13). For genome-editing therapeutics, target-tissue delivery, off-target activity, editing outcomes, and durability require additional product-specific risk assessment (5).

A directly relevant, although not yet operative, development is the Council’s September 2026 first-reading position on the revised European Union pharmaceutical Directive. The proposed framework would establish certified platform technology master files containing pre-evaluated quality, nonclinical, and clinical information and expressly identify mRNA among potential platform technologies. Reliance on such a file would remain conditional on demonstrating that the common dataset is applicable to the medicinal product and scientifically justifying product-specific customization, while the marketing authorization holder would retain responsibility for the resulting product (66). Because the Directive had not entered into force at the search cutoff, this mechanism is considered a pending legislative development rather than an available regulatory pathway.

This distinction is especially important for individualized mRNA therapeutics. Even when the manufacturing platform and LNP formulation are unchanged, the mRNA sequence and antigen combination may differ for every patient (9). Earlier platform analyses proposed defining and testing the anticipated range of patient-specific sequence variation through representative constructs and process-level controls (12, 13). For these products, the acceptable scope of sequence variation can therefore be defined through a validated algorithm, design space, manufacturing-control strategy, release-testing scheme, and traceability criteria rather than through one invariant nucleotide sequence (9, 58). MHRA’s discussion of representative batches for stability, potency or functional assays linked to biological effect, and patient-specific chain of identity—that is, continuous linkage of the patient, source material, designed sequence, manufactured batch, and intended recipient—illustrates this direction (58).

## 6 Limitations

This review has several limitations. Regulatory classifications and guidance in the jurisdictions examined continue to evolve, and some key sources are draft guidance, consultation materials, or pending legislative proposals rather than final guidance or legally operative requirements. The authorized products and development programs described in the text, including the clinical-stage candidates in **Table 2**, reflect registry, regulatory, and sponsor information available through October 13, 2026; this information is time-sensitive and is not intended as a comprehensive inventory.

## 7 Conclusion

mRNA vaccines and mRNA therapeutics share a nucleic-acid platform and certain manufacturing-process elements, but they cannot readily be treated as a single regulatory product category (1–3, 59, 60). Prophylactic mRNA vaccines express infectious-disease antigens to induce protective immune memory and can draw on accumulated regulatory experience with vaccines and biological products (6, 16). By contrast, mRNA therapeutics may express therapeutic proteins, tumor neoantigens, immunomodulatory factors, or gene-editing enzymes to treat disease, reduce recurrence, or restore function. Their evaluation must therefore place greater emphasis on target-tissue delivery, the biological activity of the expressed product, repeat administration, and long-term safety, as applicable to the individual product. Regulatory pathways should consequently be determined by the product’s intended purpose, mechanism of action, expressed product, target population, and risk profile rather than by the mRNA platform alone (9).

Taken together, the draft and consultation documents reviewed here suggest that oversight of mRNA therapeutics—particularly individualized products—is moving toward lifecycle-based management of shared platform elements and product-variable elements rather than reliance on a fixed, invariant finished-product paradigm (9, 58). For products targeting rare diseases or ultra-rare variants, large randomized trials may be infeasible; the FDA drafts discuss integrated evidence combining mechanistic plausibility, biomarkers, natural-history data, external controls, and other relevant evidence, without lowering the standards for quality, safety, and effectiveness (80, 81). Ultimately, platform-level knowledge gained from the authorization of prophylactic mRNA vaccines can support therapeutic development, but regulatory questions will vary as mRNA is applied to new types of products. Regulatory authorities in the jurisdictions reviewed here are already responding with product-specific draft guidance and consultation proposals, and these instruments are likely to change further as clinical experience accumulates. Developers will therefore need to track regulatory information across jurisdictions and engage the relevant regulatory authority early, while future regulatory frameworks combine transferable platform knowledge with product- and patient-specific risk assessment throughout the product lifecycle.
